# Estimating EQ-5D utilities based on the Short-Form Long Term Conditions Questionnaire (LTCQ-8)

**DOI:** 10.1186/s12955-020-01506-w

**Published:** 2020-08-14

**Authors:** Apostolos Tsiachristas, Caroline M. Potter, Stephen Rocks, Michele Peters, Maureen Cundell, Rupert McShane, Laurie Batchelder, Diane Fox, Julien E. Forder, Karen Jones, Felicity Waite, Daniel Freeman, Ray Fitzpatrick

**Affiliations:** 1 Health Economics Research Centre, Nuffield Department of Population Health, University of Oxford, Richard Doll Building, Old Road Campus, Oxford, OX3 7LF UK; 2 Health Services Research Unit, Nuffield Department of Population Health, University of Oxford, Oxford, UK; 3 Oxford Health NHS Foundation Trust, Oxford, UK; 4 Personal Social Services Research Unit, School of Social Policy Sociology and Social Research, University of Kent, Canterbury, UK; 5 Department of Psychiatry, University of Oxford, Oxford, UK

**Keywords:** Quality of life, EQ-5D-5L, LTCQ, Mapping, PROMs, Integrated care

## Abstract

**Purpose:**

The aim of this work was to develop a mapping algorithm for estimating EuroQoL 5 Dimension (EQ-5D) utilities from responses to the Long-Term Conditions Questionnaire (LTCQ), thus increasing LTCQ’s potential as a comprehensive outcome measure for evaluating integrated care initiatives.

**Methods:**

We combined data from three studies to give a total sample of 1334 responses. In each of the three datasets, we randomly selected 75% of the sample and combined the selected random samples to generate the estimation dataset, which consisted of 1001 patients. The unselected 25% observations from each dataset were combined to generate an internal validation dataset of 333 patients. We used direct mapping models by regressing responses to the LTCQ-8 directly onto EQ-5D-5L and EQ-5D-3L utilities as well as response (or indirect) mapping to predict the response level that patients selected for each of the five EQ-5D-5L domains. Several models were proposed and compared on mean squared error and mean absolute error.

**Results:**

A two-part model with OLS was the best performing based on the mean squared error (0.038) and mean absolute error (0.147) when estimating the EQ-5D-5L utilities. A multinomial response mapping model using LTCQ-8 responses was used to predict EQ-5D-5L responses levels.

**Conclusions:**

This study provides a mapping algorithm for estimating EQ-5D utilities from LTCQ responses. The results from this study can help broaden the applicability of the LTCQ by producing utility values for use in economic analyses.

## Introduction

In the global context of ageing populations who are likely to experience multi-morbidity, there is an increasing drive towards integrated models of care that bring together formal health services, social care provision, and community-based services to support local population needs [1, 2]. The dual aims of integrated care are to implement a person-centred approach for supporting people with complex care needs, and to ensure the sustainability of health systems over the long term. Economic evidence is an essential component of integrated care evaluation, but the evidence must be relevant to all involved stakeholders. For example in England, the economic impacts of integrated care initiatives (including pooled budgets) between Clinical Commissioning Groups responsible for health care planning and Local Authorities responsible for social care provision need to be assessed via outcomes relevant for both health and social care (e.g. well-being, independence).

Most existing outcome measures for economic evaluation, such as the EQ-5D [3], are based on the construct of Health-Related Quality of Life (HRQoL). HRQoL focuses mainly on functional status and symptom burden, for example the extent to which someone is physically mobile or experiences depression. Research highlights the limitations of this for understanding what matters to those living with long term conditions [4, 5]. Alternatively, Social Care Related Quality of Life (SCRQoL), as measured by the Adult Social Care Outcomes Toolkit (ASCOT) [6], focuses on wider aspects of well-being such as safety, personal comfort, and social participation. However, HRQoL and SCRQoL are each arguably too narrow for evaluating integrated health and social care interventions, which will simultaneously seek to minimise symptom burden (and associated use of health resources) and to maximise the efficacy of social support for ‘living well’ in its broadest sense.

The Long-Term Conditions Questionnaire (LTCQ) is a 20-item patient-reported outcome measure (PROM) designed to measure ‘living well with long-term conditions’ across health and social care domains. It enables patients to self-report on the overall impact of living with one or multiple long-term conditions (LTCs), including physical and/or mental health conditions of varying severity. LTCQ was initially validated amongst a diverse sample of health and social care users in England [7], demonstrating strong psychometric properties of internal consistency, construct validity, and test-retest reliability. It has since been validated for use in memory clinics by patients with mild cognitive impairment or dementia [8], and it is being used to evaluate clinical interventions for schizophrenia [9]. LTCQ’s person-centred construct of ‘living well’ captures outcomes that are relevant for integrated care initiatives, e.g. sense of control over daily life, safety, confidence to self-manage illness. Further psychometric analysis using Rasch modelling was undertaken to identify 8 candidate items for a Short-Form LTCQ (LTCQ-8), which correlates strongly with the 20-item version and maintains unidimensionality of its core construct [10].

The Social Care Institute for Excellence in the United Kingdom advocates three types of outcomes for evaluating integrated care: personal improvement of health and well-being (measured by PROMs), service improvement of care quality (measured by patient experience measures and process indicators), and value and sustainability of the system (e.g. cost-effectiveness and timely delivery of services to those in greatest need) [11]. A generic PROM such as EQ-5D is advantageous for its concurrent use in quality-of-life and cost-effectiveness analyses - the EQ-5D remains the National Institute for Clinical and Care Excellence’s (NICE) preferred measure of health-related quality of life [12] - but it is conceptually limited in capturing outcomes of importance for integrated care. The content of LTCQ is potentially more appropriate in this context, but LTCQ currently has no associated utility values for economic analyses. The aim of this work was to develop a mapping algorithm for estimating EQ-5D utilities from LTCQ-8 responses, thus increasing LTCQ’s potential as a comprehensive outcome measure for evaluating integrated care initiatives.

## Methods

This mapping study has been conducted following the MAPS (MApping onto Preference-based measures reporting Standards) statement [13]. The approach was also informed by ISPOR guidance on mapping from non-preference based outcome measures [14]. The valuation of the EQ-5D-5L was based on the UK value set [15] and the cross-walk to derive EQ-5D-3L utilities [16] following the latest position of National Institute for Health and Care Excellence (NICE) [12].

### Datasets

Three different datasets were used in this study. The first dataset was derived from the validation study of the LTCQ, where 1211 patients with at least one of 11 specified LTCs (cancer, chronic back pain, COPD, diabetes, depression, irritable bowel syndrome, ischemic heart disease, multiple sclerosis, osteoarthritis, severe mental health conditions including schizophrenia, stroke) were recruited from geographically diverse regions in England representing urban and rural communities, as well as areas of high and low deprivation [7]. Approximately three-quarters of the participants (health care cohort) were recruited through 15 primary care (GP) practices in 3 regions (South East, North West, Yorkshire and Humber), and the remaining quarter (social care cohort) were recruited through 4 Local Authorities (in North West, East of England, South West, and Greater London). In addition to the 20-item LTCQ participants completed several established PROMs including the EQ-5D (5-level version), the Disease Burden Impact Scale (DBIS) through which participants indicated the name and perceived impact of each long-term condition that they had, and a range of demographic questions [17]. The version of the DBIS used allows for up to 25 LTCs to be reported, each on an impact scale from 0 (does not have the condition) to 5 (high daily impact of the condition), for a theoretical maximum impact score of 125. From the 1211 participants, 37 were removed from the mapping study because of missing observations in the EQ-5D-5L questionnaire and 48 patients due to missing observations in LTCQ responses, leaving 1126 patients included in the estimation data set.

The second dataset (*n* = 115) included the baseline data from the Feeling Safe Study, a randomised controlled trial of a psychological intervention that recruited people with schizophrenia in South East England (Oxfordshire, Northamptonshire, and Berkshire) between February 2016 and July 2019 [9]. Three patients were excluded from the 118 patients in the Feeling Safe Study dataset because of missing LTCQ items. The third dataset (*n* = 93) stemmed from further validation work to test LTCQ’s psychometric properties amongst people with cognitive impairment, who were minimally represented in the original validation study. From the 102 respondents with dementia or mild cognitive impairment recruited in 14 memory clinics in South East England between February and August 2018, 9 were excluded because of missing observations in EQ-5D-5L and/or LTCQ.

In all three datasets, participants completed the full 20-item LTCQ. Responses for the 8 short-form items were extracted for the analyses below. A reduced number of LTCQ items facilitated model convergence, and these items were identified through modern psychometric methods as the best-performing, conceptually independent items for representing LTCQ’s general construct of ‘living well with long-term conditions’ [10]. As LTCQ-8 may be used as a stand-alone measure for larger-scale studies in the future, we based the mapping models on these items only (Table 1).

**Table 1:** The LTCQ-8 questionnaire

The 8 items of LTCQ-8	
4. Felt in control of daily life	
7. Felt safe at home	
8. Felt safe outside the home	
10. Felt more dependent on others than you wanted	
11. Felt lonely due to health conditions	
12. Worried about being treated differently	
15. Felt that your health conditions made you unhappy	
19. Felt confident in managing health conditions	

### Estimation dataset and internal validation

In the LTCQ validation sample, only 7% of respondents reported a severe mental health condition, and only 9 respondents reported dementia. Considering that the LTCQ is an instrument for all long-term conditions, including mental health conditions and dementia, we combined the three datasets to ensure that the estimation and validation samples for EQ-5D utilities mapping were representative of the wider LTCQ population. The inclusion of the mental health dataset allowed us to map LTCQ to low EQ-5D utilities, taking account of the major impact that severe mental conditions such as schizophrenia have on HRQoL [18, 19]. The dementia dataset was included in the estimation dataset to include the utilities of older people with affected memory problems and complex needs.

In each of the three datasets, we randomly selected 75% of the sample and combined the selected random samples to generate the estimation dataset, which consisted of 1001 patients (845 from LTCQ, 86 from mental health, 70 from dementia). The unselected 25% observations from each dataset were combined to generate an internal validation dataset of 333 patients.

### Statistical analysis

We used direct mapping models by regressing responses to the LTCQ-8 directly onto EQ-5D-5L and EQ-5D-3L utilities as well as response (or indirect) mapping to predict the response level that patients selected for each of the five EQ-5D-5L domains. The predictor variables in each fitted model were 32 dummy variables indicating whether or not a patient had a particular response on each LTCQ-8 question. We performed a complete case analysis because imputation introduces an additional source of error that can affect the precision of the algorithms [20]. Considering also the low missing data (i.e. 3%) in the estimation dataset, imputation was unlikely to affect the comparison of the mapping models. No patient characteristics (e.g. gender) were added as covariates in the best performing model because missing observations could reduce prediction accuracy. We also wanted to ensure that the mapping algorithm can be applied to datasets that do not include patient characteristics. All statistical analyses were performed in STATA version 15.

#### Direct mapping models

Ordinary least square (OLS) regression is the most frequently used mapping method [21]. However, several theoretical limitations have been highlighted in the context of HRQoL data [22]. Most particularly the assumption under OLS that the data is continuously distributed means the likelihood of having being in full health, a value of 1, is low, whereas in practice this is a relatively common response (10% of all responses considered in this study report perfect health). Despite this, recent work has shown that OLS performs well in mean prediction [23]. Tobit models have been used in EQ-5D mapping studies as an alternative to OLS to better deal with the bounded nature of EQ-5D utilities [24, 25]. Thus, the lower and upper limits for a Tobit model were specified as the minimum and maximum possible score on the EQ-5D-5L scale (i.e. -0.285 and 1 respectively) and on the EQ-5D-3L scale (i.e. -0.594 and 1 respectively). In the presence of heteroscedasticity, non-normality, and censoring, censored least absolute deviations (CLAD) models provide consistent estimates [23] and thus, it has been used extensively in HRQoL mapping literature [21, 26]. The limits and dependent variable in this model were specified as in the Tobit model. The observed EQ-5D-5L utility was used as the dependent variable in the OLS, Tobit and CLAD models.

Generalised Linear Model (GLM) allows for the skewed distribution of EQ-5D utility data and prevents prediction of utilities higher than 1 [27]. We specified two GLMs, one with Gamma family and log link and another with Gaussian family and log link, to predict EQ-5D-5L disutility. To fit these models, the dependent variable was transformed as 1 − *Utility*. Furthermore, fractional logistic regression was used to constrain utility predictions between − 0.281 and 1 for EQ-5D-5L utilities, and between − 0.594 and 1 for EQ-5D-3L utilities. The dependent variable in the regression with the EQ-5D-5L utility was

1 $$ {Utility}_{0-1}=\left( Utility+0.285\right)/1.285 $$

Two-part models have been used in utility mapping studies to allow for a relatively large proportion of observations reporting perfect health on EQ-5D (i.e. utility of 1) [22]. We therefore, specified two two-part models. In the first part of these models, a logistic regression was used to estimate the probability an individual to report perfect health (i.e. EQ-5D-5L utility = 1). The second part applied OLS regression in one two-part model and binomial beta regression in another two-part model to utilities less than 1. Binomial beta regression was performed because of its ability to deal with left and right skewed HRQoL data [28]. The dependent variable in the binomial beta regression was specified similar to the fractional logistic regression.

#### Response mapping models

Several models have been proposed in the literature to predict responses to EQ-5D dimensions [22], the most frequently used are multinomial and ordinal logistic regressions [21]. A multinomial logit and an ordinal logit were specified to estimate responses based on the expected value method [22, 29]. These regression models were applied to each dimension of the EQ-5D-5L and used to calculate the probabilities of responding to each of the five levels. The expected dis-utilities (inverse of utility) in each dimension were calculated by using the estimated response probabilities and the EQ-5D-5L UK valuation set [15].

### Assessment of model performance

Predicted EQ-5D-5L utilities were estimated for each mapping model with back-transformation applied to the GLM models, fractional logistic regression, and binomial beta regression. For the two-part models, expected utility was estimated as:

2 $$ Utility=\Pr \left( Utility=1\right)+\Big(1-\Pr \left( Utility=1\right)\ast U $$

where *U* is the utility conditional on imperfect health (i.e. < 1) estimated in the second part of the models.

The mapping models were compared in terms of their ability to accurately predict EQ-5D-5L utility based on common metrics of predictive performance. Mean squared error (MSE) indicated goodness-of-fit and mean absolute error (MAE) measured individual-level prediction accuracy. These metrics were calculated in the estimation sample as well as in the two external validation models separately and jointly. Scatterplots with the observed and predicted EQ-5D-5L and EQ-5D-3L utility were created for each mapping model.

After the best performing model was selected, we investigated whether it performed equally well across different levels of morbidity and between males and females. This was done by plotting MSE across the deciles of the DBIS Score by gender. This was done using only the original LTCQ validation dataset because the DBIS Score was not available in the other two datasets.

## Results

The mean EQ-5D-5L utility in the dataset from the validation study of the LTCQ was 0.616 (SD: 0.320). A similar mean utility was recorded in the baseline dataset from the Feeling Safe study among a sample with mental health conditions (mean: 0.605, SD: 0.219) and a higher utility recorded in the sample with dementia or mild cognitive impairment from the additional validation of the LTCQ (mean: 0.785, SD: 0.205). In our combined estimation sample of 1001 participants from across the datasets, the mean score was 0.623 (SD: 0.312). The mean DBIS score, only recorded for the full LTCQ validation sample, was 15.936 (SD: 12.814).

**Fig. 1:**
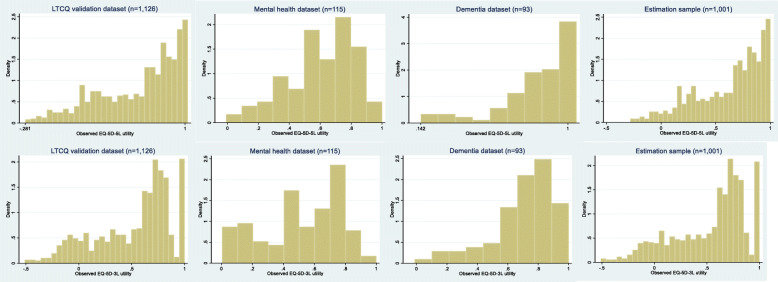
Distribution of EQ-5D-5L and EQ-5D-3L (using crosswalk) utility in each dataset

Table 2 provides the responses for the EQ-5D-5L and LTCQ-8 questions by dataset. Figure 1 provides the distribution of scores on the EQ-5D-5L and the EQ-5D-3L by dataset. This illustrates the bounded and skewed distribution of the utilities.

**Table 2:** Responses on EQ-5D-5L and LTCQ-8 questions by dataset

Model	LTCQ validation dataset (*n* = 1126)	Mental health dataset (*n* = 115)	Dementia dataset (*n* = 93)	Estimation dataset (*n* = 1001)
EQ-5D Mobility				
No Problems	367 (32%)	61 (53%)	48 (52%)	360 (36%)
Slight Problems	202 (18%)	23 (20%)	13 (14%)	176 (18%)
Moderate Problems	223 (20%)	20 (17%)	24 (26%)	200 (20%)
Severe problems	231 (21%)	10 (9%)	8 (8%)	185 (19%)
Unable	103 (9%)	1 (1%)	0 (0%)	80 (8%)
EQ-5D Self-care				
No Problems	655 (58%)	62 (54%)	71 (77%)	593 (59%)
Slight Problems	154 (14%)	23 (20%)	13 (14%)	142 (14%)
Moderate Problems	144 (13%)	23 (20%)	3 (3%)	126 (13%)
Severe problems	83 (7%)	7 (6%)	4 (4%)	68 (7%)
Unable	90 (8%)	0 (0%)	2 (2%)	72 (7%)
EQ-5D Usual activities				
No Problems	352 (31%)	18 (16%)	50 (54%)	311 (31%)
Slight Problems	236 (21%)	27 (24%)	17 (18%)	206 (21%)
Moderate Problems	245 (22%)	42 (37%)	17 (18%)	232 (23%)
Severe problems	152 (14%)	22 (19%)	5 (6%)	135 (13%)
Unable	141 (12%)	5 (5%)	4 (4%)	117 (12%)
EQ-5D Pain/Discomfort				
No Problems	258 (23%)	38 (33%)	42 (45%)	256 (25%)
Slight Problems	331 (29%)	31 (27%)	22 (24%)	290 (29%)
Moderate Problems	294 (26%)	33 (28%)	25 (27%)	266 (26%)
Severe problems	183 (16%)	12 (10%)	3 (3%)	146 (15%)
Unable	60 (5%)	1 (1%)	1 (1%)	43 (4%)
EQ-5D Anxiety/depression Anxiety/Depression				
No Problems	471 (42%)	5 (4%)	37 (40%)	396 (40%)
Slight Problems	301 (27%)	17 (15%)	32 (34%)	247 (25%)
Moderate Problems	232 (20%)	49 (43%)	20 (22%)	224 (22%)
Severe problems	77 (7%)	29 (25%)	4 (4%)	90 (9%)
Unable	45 (4%)	15 (13%)	0 (0%)	44 (4%)
LTCQ4. Control of daily life				
Never	94 (8%)	9 (8%)	3 (3%)	83 (8%)
Rarely	142 (13%)	51 (44%)	9 (10%)	140 (14%)
Sometimes	242 (21%)	36 (31%)	22 (24%)	232 (23%)
Often	236 (21%)	19 (17%)	17 (18%)	205 (20%)
Always	412 (37%)	0 (0%)	42 (45%)	341 (34%)
LTCQ7. Safe at home				
Never	19 (2%)	4 (4%)	2 (2%)	18 (2%)
Rarely	38 (3%)	24 (21%)	1 (1%)	49 (5%)
Sometimes	109 (10%)	43 (37%)	3 (3%)	120 (12%)
Often	212 (19%)	31 (27%)	14 (15%)	188 (19%)
Always	748 (66%)	13 (11%)	73 (79%)	626 (62%)
LTCQ8. Safe outside home				
Never	95 (8%)	20 (17%)	7 (8%)	86 (8%)
Rarely	116 (11%)	44 (38%)	7 (8%)	128 (13%)
Sometimes	251 (22%)	35 (30%)	15 (16%)	227 (23%)
Often	212 (19%)	10 (9%)	20 (21%)	174 (17%)
Always	452 (40%)	6 (5%)	44 (47%)	386 (38%)
LTCQ10. Dependant				
Always	292 (26%)	17 (15%)	12 (13%)	242 (24%)
Often	224 (20%)	49 (43%)	28 (30%)	228 (23%)
Sometimes	226 (20%)	32 (28%)	25 (27%)	215 (21%)
Rarely	155 (14%)	13 (11%)	16 (17%)	131 (13%)
Never	229 (20%)	4 (3%)	2 (13%)	185 (18%)
LTCQ11. Lonely				
Always	126 (11%)	39 (34%)	5 (5%)	133 (13%)
Often	162 (14%)	40 (35%)	11 (12%)	155 (15%)
Sometimes	264 (23%)	24 (21%)	20 (22%)	233 (23%)
Rarely	167 (15%)	4 (4%)	15 (16%)	131 (13%)
Never	407 (36%)	8 (7%)	42 (45%)	349 (35%)
LTCQ12. Stigma				
Always	77 (7%)	28 (24%)	3 (3%)	77 (8%)
Often	116 (10%)	44 (38%)	21 (23%)	121 (12%)
Sometimes	281 (25%)	30 (26%)	22 (24%)	243 (24%)
Rarely	198 (18%)	10 (9%)	47 (50%)	182 (18%)
Never	454 (40%)	3 (3%)	0 (0%)	378 (38%)
LTCQ15. Unhappy				
Always	148 (13%)	31 (27%)	4 (4%)	139 (14%)
Often	203 (18%)	48 (42%)	14 (15%)	195 (19%)
Sometimes	350 (31%)	28 (24%)	33 (35%)	301 (30%)
Rarely	185 (17%)	5 (4%)	22 (24%)	164 (16%)
Never	240 (21%)	3 (3%)	20 (22%)	202 (20%)
LTCQ19. Confident				
Never	59 (5%)	5 (4%)	6 (6%)	53 (5%)
Rarely	84 (8%)	27 (24%)	2 (2%)	82 (8%)
Sometimes	254 (23%)	60 (52%)	17 (18%)	252 (25%)
Often	252 (22%)	18 (16%)	25 (26%)	223 (22%)
Always	477 (42%)	5 (4%)	43 (46%)	391 (39%)

### Direct mapping

Table 3 shows the performance of the various models when estimating EQ-5D-5L and EQ-5D-3L utilities within the estimation dataset. The two-part model with OLS was the best performing model based on the mean squared error (0.038) and mean absolute error (0.147) when estimating the EQ-5D-5L, as well for EQ-5D-3L utilities (MSE: 0.052, MAE: 0.172). The order of rank was largely consistent based on either the mean squared error or mean absolute error and between utilities. The mean squared error was consistently lower for the 5 L utility compared to the 3 L. Of the possible response mapping models, the multinomial logit was the better performing (MSE: 0.045; MAE: 0.155).

**Table 3:** Model performance based on the estimation sample (*n* = 1001)

	EQ-5D-5L utility	Model rank		EQ-5D-3L utility	Model rank	
Model	MSE (MAE)	MSE	MAE	MSE (MAE)	MSE	MAE
1. OLS	0.0390 (0.1486)	2	2	0.0525 (0.1732)	2^a^	3
2. Tobit	0.0397 (0.1490)	4	3	0.0536 (0.1744)	5	5
3. CLAD	0.0436 (0.1566)	7	9	0.0574 (0.1808)	7	7
4. GLM (Gamma; Log link)	0.0414 (0.1515)	6	5	0.0540 (0.1742)	6	4
5. GLM (Gaussian; Log link)	0.0399 (0.1527)	5	6	0.0533 (0.1747)	4	6
6. Fractional logit	0.0392 (0.1492)	3	4	0.0525 (0.1724)	2^a^	2
7. Two-part model with OLS	0.0384 (0.1474)	1	1	0.0516 (0.1716)	1	1
8. Two-part model with beta regression	0.0473 (0.1575)	10	10	0.0903 (0.2157)	10	10
9. Multinomial logit	0.0451 (0.1550)	8	7	0.0675 (0.1972)	8	8
10. Ordered logit	0.0458 (0.1563)	9	8	0.0705 (0.1999)	9	9

*OLS* Ordinary Least Square, *GLM* Generalised Linear Model, *MSE* Mean Squared Error, *MAE* Mean Absolute Error

^a^tied

**Fig. 2:**
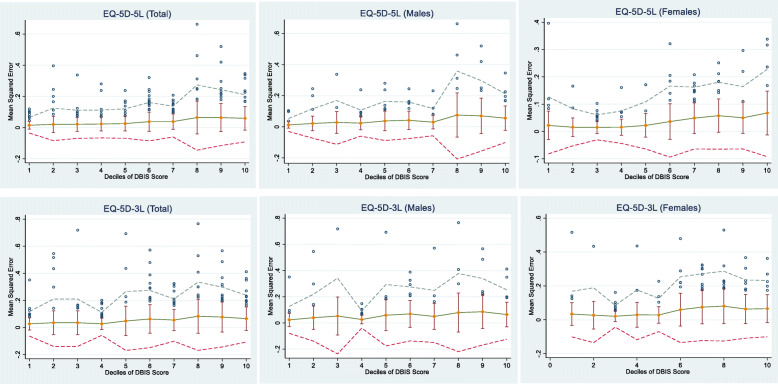
Mean Squared Errors across deciles of the DBIS score by gender based on the LTCQ validation dataset (*n* = 1126). Notes: Bars show the standard deviation of the mean Mean Squared Error in each decile; Dashed lines show the outer fence of 2 standard deviations from the mean Mean Squared Error in each decile; Dots show outliers with Mean Squared Error larger than one Standard Deviation of the mean Mean Squared Error in each decile

Figure 2 shows the performance of the two-part OLS model as measured by the mean squared error across deciles of the DBIS scores. This confirms, as per Table 3, that the model performs better on the EQ-5D-5L compared to the 3 L. It also shows that model performance deteriorates among those with higher DBIS scores, representing high severity and/or multiple conditions, especially among males.

Table 4 provides regression coefficients for the best performing model, the two-part model with OLS. The results can be used to estimate the EQ-5D utilities based on a set of responses to the Short-Form LTCQ according to Eq. 2:

2 $$ Utility=\Pr \left( Utility=1\right)+\Big(1-\Pr \left( Utility=1\right)\ast U $$

**Table 4:** Regression coefficients for the best model: two-part with OLS

		EQ 5D-5L utilities	EQ 5D-3L utilities using crosswalk
	Part 1- Logit	Part 2- OLS	Part 2- OLS
*Reference category in brackets*	Odds Ratio (se)	b (se)	b (se)
LTCQ4. Control of daily life (Never)			
Rarely	1.240 (2.0123)	0.167 (0.030)*	0.220 (0.034)*
Sometimes	−0.448 (2.091)	0.174 (0.030)*	0.233 (0.034)*
Often	−0.045 (1.866)	0.209 (0.034)*	0.273 (0.039)*
Always	1.145 (1.831)	0.235 (0.037)*	0.286 (0.042)*
LTCQ7. Safe at home (Never)			
Rarely	−31.701 (3435.388)	0.017 (0.060)	0.027 (0.068)
Sometimes	−31.103 (3295.443)	−0.016 (0.056)	− 0.017 (0.064)
Often	−16.537 (1488.793)	−0.031 (0.056)	− 0.033 (0.064)
Always	−17.518 (1488.793)	0.014 (0.056)	−0.010 (0.064)
LTCQ8. Safe outside home (Never)			
Rarely	−0.674 (2939.661)	0.013 (0.031)	0.010 (0.035)
Sometimes	14.568 (1488.792)	0.191 (0.023)	0.075 (0.034)*
Often	13.895 (1488.792)	0.225 (0.028)*	0.109 (0.038)*
Always	15.067 (1488.792)	0.235 (0.029)*	0.166 (0.039)*
LTCQ10. Dependant (Always)			
Often	−0.738 (0.842)	0.138 (0.021)*	0.162 (0.024)*
Sometimes	−0.483 (0.611)	0.191 (0.023)*	0.221 (0.026)*
Rarely	0.070 (0.616)	0.225 (0.028)*	0.258 (0.032)*
Never	0.263 (0.587)	0.235 (0.029)*	0.268 (0.033)*
LTCQ11. Lonely (Always)			
Often	0.015 (1.017)	−0.028 (0.028)	−0.016 (0.031)
Sometimes	−1.439 (0.920)	−0.035 (0.028)	−0.030 (0.032)
Rarely	−0.915 (0.814)	−0.015 (0.032)	− 0.006 (0.036)
Never	−0.410 (0.700)	− 0.024 (0.031)	− 0.016 (0.036)
LTCQ12. Stigma (Always)			
Often	−26.818 (2169.424)	−0.017 (0.032)	−0.002 (0.036)
Sometimes	−0.516 (1.373)	− 0.033 (0.031)	− 0.009 (0.035)
Rarely	−0.737 (1.366)	0.006 (0.033)	0.020 (0.037)
Never	−0.845 (1.337)	−0.034 (0.033)	− 0.020 (0.038)
LTCQ15. Unhappy (Always)			
Often	16.502 (2105.194)	0.084 (0.025)*	0.084 (0.028)*
Sometimes	17.163 (2105.194)	0.149 (0.027)*	0.138 (0.031)*
Rarely	18.324 (2105.194)	0.155 (0.032)*	0.121 (0.037)*
Never	18.952 (2105.194)	0.152 (0.033)*	0.140 (0.039)*
LTCQ19. Confident (Never)			
Rarely	−15.296 (3063.957)	0.020 (0.038)	0.014 (0.043)
Sometimes	−1.553 (1.622)	0.018 (0.035)	0.034 (0.040)
Often	−0.167 (1.169)	0.034 (0.038)	0.062 (0.043)
Always	0.315 (1.082)	0.070 (0.039)	0.120 (0.044)*
Constant	−16.994 (2105.193)	0.061 (0.058)	−0.130 (0.066)*
n	1001	896	896

*SE* standard error

* *p*-value< 0.005

For instance, in a set of responses for an individual responding “living well” throughout the LTCQ, the probability of being in perfect health (utility of 1) is given by the sum of the constant and the coefficients for “living well” across each domain for part 1 – note, this needs to be transformed by taking the inverse of the logistic function. The predicted second-part utility is given by the sum of the constant and respective domain coefficients from the part 2 estimates. Finally, the predicted EQ-5D utility is given by substituting the above values into Eq. 2, producing a utility estimate of 0.93 for the EQ-5D-5L (see Appendix 2 for a detailed calculation).

### Out-of-sample prediction

When we fitted the coefficients to the validation sample (*n* = 333), the mean predicted EQ-5D-5L utility from the two-part OLS model was 0.64 (SD: 0.25), compared to the recorded mean of 0.63 (SD: 0.30). The mean predicted EQ-5D-3L was 0.55 (SD: 0.28), compared to a recorded 0.53 (SD: 0.34). The mean squared error of our predicted value was 0.040 for the EQ. 5D-5L and 0.0582 for the EQ. 5D-3L; the mean absolute error was 0.1520 and 0.1848, respectively.

### Response mapping

Appendix 3 reports the multinomial logit results for each of the EQ-5D questions with the 8 items of the short-form LTCQ as the predictor variables. The McFadden pseudo-R2 indicates that the models perform fairly well, particularly in the self-care, usual activity and anxiety/depression domains. The results were broadly intuitive. In several instances, the result for a given LTCQ-8 question most related to an EQ-5D domain is significant; for example, the LTCQ-8 question on feeling dependent is significant across a number of EQ-5D domains but particularly for usual activities and less so for anxiety and depression.

## Discussion

This study provides a mapping algorithm for estimating EQ-5D utilities from LTCQ-8 responses. We have shown that our approach produces estimated utility scores that closely approximate what would have been recorded had the EQ-5D been administered, particularly for individuals outside of the most severe health states. The results from this study can help extend the applicability of the LTCQ by estimating utility values for use in economic analyses.

In this paper, we presented a broad range of potential models. Consistent with previous studies, a simple OLS model performed well for predicting average utilities, but the two-part model with OLS emerged as the best-performing model. The two-part model was proposed to better reflect the bounded nature of EQ-5D scores. The mean squared errors and mean absolute errors recorded in our models were similar to that reported elsewhere [30]. The small differences in performance between several models is consistent with research elsewhere [22, 31], as well as with the finding that the validity of mapping algorithms is more strongly a function of the particular instrument and disease or patient group rather than the specific technique employed [32].

We mapped our results onto both EQ-5D-5L and EQ-5D-3L utilities. The NICE position statement from November 2018 affirms the present need to use the EQ-5D-3L for reference-case analyses and recommends mapping to the EQ-5D-3L if EQ-5D-5L data had been collected [12]. EQ-5D-3L values in this study were thus derived using the recommended crosswalk [16]. Our models performed better on the EQ. 5D-5L than the 3 L, implying that the greater sensitivity afforded by the additional levels in the EQ-5D-5L helped achieve improved model fit.

In addition, we mapped a response model (multinomial logit) that indicates how responses to questions from the LTCQ-8 impact across the dimensions of the EQ-5D-5L. This mainly provided intuitive results, with responses to particular questions affecting appropriate domains. For instance, feeling more in control of daily life was significantly associated with being in a less severe state in the mobility domain. Although, there were also some seemingly counter-intuitive signs, for instance ‘always feeling safe at home’ was a positive and significant predictor of being in the most severe state in the mobility domain relative to the least severe. Albeit, this could reflect the person being in adjusted accommodation where they do feel safe. A larger validation dataset would help to clarify this.

Similarly, a validation dataset with more severe health states (lower utilities) would help assess model performance where it was weakest, although worsening predictive performance for patients in severe states is consistent with other research [30]. Similarly, since utilities are often required for specific subgroups of patients, further validation could occur with populations dissimilar from those featured in this paper.

## Conclusions

As a result of the mapping algorithm produced in this study, surveys that include the LTCQ, or the short form LTCQ-8, can now be used to estimate utility values for economic analyses. Although the existence of a mapping algorithm is not an argument against the inclusion of direct preference-based measures in prospective studies - mapping is a second-best solution to direct measurement [33] - the ability to generate estimated utilities should increase the potential of the LTCQ for use as a comprehensive outcome measure for evaluating integrated care initiatives.

## Figures and Tables

**Fig. 3:**
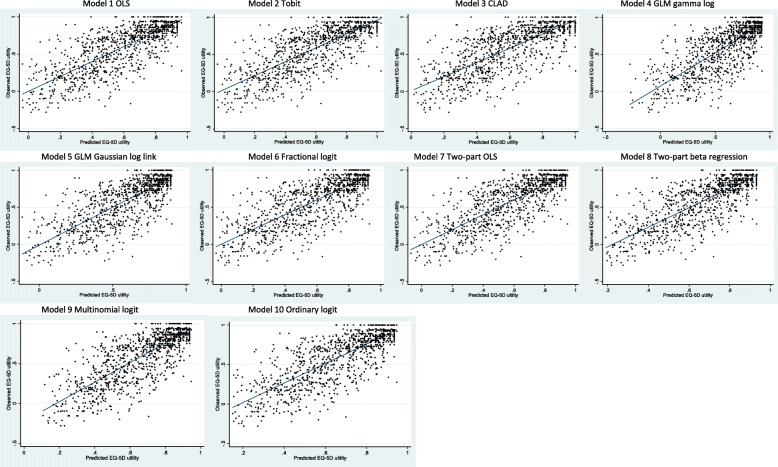
Scatter plots of the observed EQ-5D-5L utility versus predicted EQ-5D-5 L utility

## Data Availability

The datasets generated analysed during the current study are not publicly available due restricting data sharing agreements in the three studies that provided data but might be available from the corresponding author on reasonable request.

## Appendix 2

### Estimating utilities based on responses to the short form LTCQ

EQ 5D utilities can be estimated for the best-fitting model according to Eq. 2:

2 $$ Utility=\Pr \left( Utility=1\right)+\Big(1-\Pr \left( Utility=1\right)\ast U $$

Based on Table 3, the utility for someone responding “living well” throughout the survey is estimated as follows:

Part one estimate

To estimate the probability of being in perfect health, we sum the constant and domain coefficients and use the inverse of the logistic function to re-transform the data. The inverse logistic function is *exp*(*x*)/(1 +  *exp* (*x*)) and can be calculated using the Stata command “invlogit”.

$$ \Pr \left( Utility=1\right)= InvLogit\Big(\_b\left[ Constant\right]+\_b\left[ Control\right]+\_b\left[ Safe\  at\  home\right]+\_b\left[ Safe\ outs\mathrm{i} de\  home\right]+\_b\left[ Dependant\right]+\_b\left[ Lonely\right]+\_b\left[ Stigma\right]+\_b\left[ Unhappy\right]+\_b\left[ Confident\right] $$

Pr(*Utility* = 1) = *InvLogit*( − 17.0 + 1.1 + 17.2 + 15.1 + 0.3 ± 0.4 ± 0.8 + 18.6 + 0.3) = 0.49.

Part two estimate

$$ U=\Big(\_b\left[ Constant\right]+\_b\left[ Control\right]+\_b\left[ Safe\  at\  home\right]+\_b\left[ Safe\ outside\ home\right]+\_b\left[ Dependant\right]+\_b\left[ Lonely\right]+\_b\left[ Stigma\right]+\_b\left[ Unhappy\right]+\_b\left[ Confident\right] $$

*U* = (0.06 + 0.24 + 0.01 + 0.16 + 0.23 +  − 0.02 +  − 0.03 + 0.15 + 0.07) = 0.87

Overall utility estimate

*Utility* = Pr(*Utility* = 1) + (1 − Pr(*Utility* = 1) ∗ *U* =0.49 + ((1 − 0.49) ∗ 0.87) = 0.93

## Appendix 3

**Table 5:** Response mapping model of LTCQ fitted using multinomial logistic regression

L1 (No Problems)	L2 vs L1	L3 vs L1	L4 vs L1	L5 vs L1
	Slight Problems	Moderate Problems	Severe Problems	Unable
**Mobility**				
LTCQ4. Control of daily life (Never)				
Rarely	0.669 (0.913)	−0.779 (0.635)	−1.97 (0.591)*	−2.85 (0.664)*
Sometimes	0.92 (0.917)	−0.358 (0.638)	−1.542 (0.598)*	−2.064 (0.652)*
Often	1.253 (0.935)	−0.981 (0.677)	−1.581 (0.645)*	−2.67 (0.762)*
Always	0.9 (0.944)	−1.407 (0.705)*	−2.441 (0.696)*	−2.752 (0.811)*
LTCQ7. Safe at home (Never)				
Rarely	1.376 (1.277)	1.058 (1.002)	−0.488 (0.858)	−0.718 (1.168)
Sometimes	1.158 (1.252)	1.065 (0.978)	0.396 (0.796)	1.181 (1.025)
Often	1.718 (1.245)	1.381 (0.98)	1.073 (0.801)	2.057 (1.036)*
Always	1.713 (1.248)	1.769 (0.982)	0.526 (0.806)	2.121 (1.038)*
LTCQ8. Safe outside home (Never)				
Rarely	−0.357 (0.671)	−0.488 (0.578)	− 0.689 (0.562)	− 0.976 (0.66)
Sometimes	− 0.429 (0.641)	− 0.628 (0.567)	−1.457 (0.556)*	−1.449 (0.639)*
Often	− 0.927 (0.684)	−1.095 (0.615)	−2.633 (0.632)*	−1.611 (0.727)*
Always	−1.544 (0.679)*	−2.213 (0.625)*	−3.044 (0.626)*	−3.306 (0.754)*
LTCQ10. Dependant (Always)				
Often	0.405 (0.419)	0.226 (0.339)	−1.177 (0.335)*	−1.791 (0.439)*
Sometimes	0.582 (0.411)	−0.182 (0.35)	−1.599 (0.362)*	−2.667 (0.551)*
Rarely	0.349 (0.435)	−1.099 (0.424)*	−3.279 (0.608)*	−3.424 (0.829)*
Never	−0.197 (0.448)	−2.724 (0.548)*	−3.998 (0.654)*	−4.935 (1.116)*
LTCQ11. Lonely (Always)				
Often	0.38 (0.464)	0.379 (0.402)	0.685 (0.432)	−0.26 (0.567)
Sometimes	0.574 (0.463)	0.281 (0.42)	1.205 (0.445)*	0.684 (0.542)
Rarely	0.69 (0.501)	0.504 (0.468)	1.534 (0.516)*	0.871 (0.654)
Never	0.778 (0.478)	0.733 (0.447)	1.388 (0.512)*	0.974 (0.651)
LTCQ12. Stigma (Always)				
Often	−0.362 (0.554)	0.207 (0.509)	0.416 (0.501)	0.661 (0.604)
Sometimes	−0.429 (0.533)	0.414 (0.494)	0.915 (0.487)	0.709 (0.585)
Rarely	−0.495 (0.55)	0.373 (0.516)	0.154 (0.537)	0.352 (0.655)
Never	0.028 (0.55)	0.884 (0.530)	1.142 (0.550)*	0.688 (0.668)
LTCQ15. Unhappy (Always)				
Often	−0.218 (0.46)	−0.392 (0.400)	− 0.465 (0.408)	− 0.397 (0.509)
Sometimes	0.182 (0.474)	0.139 (0.425)	−0.674 (0.449)	− 0.443 (0.577)
Rarely	−0.363 (0.524)	−0.42 (0.510)	− 0.49 (0.544)	0.649 (0.694)
Never	−0.186 (0.527)	0.238 (0.509)	−0.377 (0.577)	− 0.332 (0.829)
LTCQ19. Confident (Never)				
Rarely	−1.282 (0.748)	−0.628 (0.722)	− 0.475 (0.722)	− 0.215 (0.788)
Sometimes	− 1.711 (0.691)*	− 0.823 (0.681)	− 0.066 (0.684)	− 0.415 (0.75)
Often	− 1.588 (0.71)*	− 0.564 (0.708)	0.256 (0.721)	−1.098 (0.834)
Always	−1.866 (0.706)*	− 1.147 (0.718)	− 0.143 (0.739)	− 1.011 (0.843)
Constant	− 1.031 (1.428)	0.56 (1.123)	2.796 (0.947)*	2.424 (1.114)*
**Self-care**				
LTCQ4. Control of daily life (Never)				
Rarely	−1.097 (0.664)	−1.309 (0.615)*	− 1.970 (0.640)*	− 2.689 (0.643)*
Sometimes	− 1.038 (0.653)	− 1.539 (0.615)*	− 2.239 (0.640)*	− 2.919 (0.649)*
Often	− 1.354 (0.688)*	− 2.537 (0.691)*	− 2.767 (0.776)*	−3.368 (0.735)*
Always	− 1.889 (0.757)*	− 2.055 (0.732)*	− 4.186 (1.037)*	−3.548 (0.819)*
LTCQ7. Safe at home (Never)				
Rarely	−0.416 (0.878)	1.185 (0.957)	0.423 (1.092)	−0.113 (1.172)
Sometimes	− 0.269 (0.812)	0.209 (0.928)	1.47 (0.993)	0.877 (1.052)
Often	−0.345 (0.811)	0.988 (0.91)	1.347 (1.006)	1.065 (1.058)
Always	−0.558 (0.815)	0.733 (0.918)	1.462 (1.004)	0.914 (1.058)
LTCQ8. Safe outside home (Never)				
Rarely	0.297 (0.546)	−0.653 (0.500)	−0.335 (0.579)	− 0.391 (0.592)
Sometimes	0.151 (0.527)	−0.936 (0.478)*	−0.878 (0.568)	− 0.917 (0.567)
Often	−0.593 (0.578)	− 0.914 (0.531)	−1.362 (0.750)	− 0.822 (0.654)
Always	−1.335 (0.616)*	−2.352 (0.605)*	−1.828 (0.740)*	− 1.902 (0.680)*
LTCQ10. Dependant (Always)				
Often	0.074 (0.309)	−0.534 (0.307)	−1.301 (0.420)*	−1.699 (0.421)*
Sometimes	−0.347 (0.331)	−1.345 (0.365)*	−1.479 (0.489)*	−2.661 (0.596)*
Rarely	−1.176 (0.460)*	−3.128 (0.783)*	−15.851 (619.704)	−2.987 (0.817)*
Never	−2.646 (0.803)*	−3.201 (0.831)*	−2.136 (0.864)*	−4.182 (1.131)*
LTCQ11. Lonely (Always)				
Often	−0.138 (0.4)	0.817 (0.441)	−0.068 (0.543)	0.053 (0.556)
Sometimes	0.048 (0.409)	0.775 (0.466)	0.665 (0.538)	0.243 (0.554)
Rarely	−0.014 (0.464)	−0.013 (0.569)	− 0.123 (0.705)	0.160 (0.659)
Never	0.138 (0.476)	0.974 (0.538)	−0.504 (0.806)	0.652 (0.662)
LTCQ12. Stigma (Always)				
Often	0.675 (0.495)	0.532 (0.503)	−0.159 (0.575)	0.291 (0.601)
Sometimes	0.56 (0.487)	0.345 (0.491)	0.046 (0.544)	0.418 (0.570)
Rarely	0.373 (0.515)	0.212 (0.528)	−1.226 (0.730)	0.081 (0.648)
Never	0.658 (0.533)	0.014 (0.564)	−0.507 (0.693)	0.163 (0.661)
LTCQ15. Unhappy (Always)				
Often	−0.385 (0.375)	−0.412 (0.391)	− 0.269 (0.477)	0.087 (0.488)
Sometimes	−0.258 (0.399)	−0.151 (0.426)	0.026 (0.542)	−0.026 (0.549)
Rarely	−0.002 (0.504)	−0.056 (0.586)	1.027 (0.764)	0.884 (0.705)
Never	0.06 (0.542)	0.029 (0.610)	1.292 (0.883)	−1.618 (1.190)
LTCQ19. Confident (Never)				
Rarely	0.67 (0.818)	−0.402 (0.676)	−0.049 (0.708)	− 0.686 (0.737)
Sometimes	0.446 (0.771)	−0.615 (0.617)	−1.111 (0.676)	−1.128 (0.673)
Often	0.35 (0.795)	−0.882 (0.651)	−1.97 (0.798)*	− 1.176 (0.73)
Always	−0.016 (0.821)	−1.121 (0.679)	− 1.887 (0.822)*	− 0.986 (0.767)
Constant	0.613 (1.095)	1.801 (1.044)	2.362 (1.072)*	2.893 (1.119)*
**Usual activities**				
LTCQ4. Control of daily life (Never)				
Rarely	1.007 (1.102)	1.22 (0.964)	−0.325 (0.888)	−1.517 (0.894)
Sometimes	0.842 (1.015)	0.869 (0.882)	−1.009 (0.801)	−2.246 (0.811)*
Often	0.262 (1.018)	0.201 (0.893)	−1.655 (0.829)*	−3.144 (0.882)*
Always	−0.022 (1.022)	− 0.538 (0.911)	−3.052 (0.906)*	− 3.369 (0.903)*
LTCQ7. Safe at home (Never)				
Rarely	0.87 (1.439)	1.434 (1.29)	1.463 (1.322)	1.116 (1.394)
Sometimes	0.972 (1.262)	1.251 (1.126)	1.524 (1.147)	2.322 (1.195)
Often	0.756 (1.226)	1.075 (1.09)	1.978 (1.111)	2.527 (1.167)*
Always	0.486 (1.216)	0.856 (1.077)	1.512 (1.099)	2.432 (1.153)*
LTCQ8. Safe outside home (Never)				
Rarely	0.606 (1.05)	1.295 (0.985)	0.395 (0.986)	0.094 (1.004)
Sometimes	−0.431 (0.778)	−0.256 (0.719)	−1.34 (0.718)	−1.631 (0.74)*
Often	0.009 (0.801)	−0.389 (0.751)	−1.811 (0.781)*	−2.028 (0.835)*
Always	−0.736 (0.789)	−1.309 (0.74)	− 2.896 (0.788)*	−3.197 (0.804)*
LTCQ10. Dependant (Always)				
Often	0.645 (0.462)	0.516 (0.432)	−0.308 (0.457)	−1.145 (0.497)*
Sometimes	0.276 (0.42)	− 0.096 (0.394)	−1.372 (0.469)*	−2.302 (0.571)*
Rarely	−0.123 (0.446)	−1.684 (0.481)*	−2.200 (0.603)*	−3.700 (1.091)*
Never	−0.562 (0.461)	−2.276 (0.550)*	− 1.996 (0.650)*	−3.221 (0.811)*
LTCQ11. Lonely (Always)				
Often	0.097 (0.588)	−0.419 (0.53)	0.328 (0.581)	0.340 (0.634)
Sometimes	1.097 (0.561)	0.234 (0.517)	1.015 (0.584)	1.218 (0.629)
Rarely	1.349 (0.587)*	0.4 (0.55)	0.152 (0.724)	0.931 (0.728)
Never	1.289 (0.566)*	0.627 (0.521)	1.732 (0.641)*	0.806 (0.735)
LTCQ12. Stigma (Always)				
Often	0.307 (0.698)	−0.466 (0.646)	0.432 (0.688)	−0.258 (0.722)
Sometimes	0.742 (0.662)	0.629 (0.605)	1.159 (0.663)	0.647 (0.682)
Rarely	0.529 (0.67)	0.592 (0.624)	0.556 (0.713)	0.692 (0.737)
Never	0.38 (0.665)	0.948 (0.615)	0.989 (0.719)	0.966 (0.751)
LTCQ15. Unhappy (Always)				
Often	−0.507 (0.633)	−0.286 (0.597)	− 0.469 (0.622)	− 0.388 (0.658)
Sometimes	− 0.824 (0.604)	− 1.037 (0.578)	− 1.341 (0.621)*	− 1.208 (0.676)
Rarely	− 1.262 (0.631)*	− 1.631 (0.632)*	− 1.780 (0.741)*	− 1.004 (0.81)
Never	− 1.654 (0.640)*	− 1.415 (0.631)*	−2.019 (0.770)*	− 1.320 (0.865)
LTCQ19. Confident (Never)				
Rarely	0.496 (1.162)	0.221 (0.974)	0.144 (1.003)	0.283 (0.993)
Sometimes	0.950 (1.014)	0.087 (0.825)	0.573 (0.86)	−0.361 (0.863)
Often	0.860 (1.014)	0.145 (0.826)	0.45 (0.879)	−0.633 (0.9)
Always	0.323 (1.003)	−0.675 (0.814)	− 0.342 (0.881)	−1.223 (0.894)
Constant	−1.638 (1.561)	0.300 (1.272)	1.448 (1.218)	2.872 (1.221)*
**Pain and discomfort**				
LTCQ4. Control of daily life (Never)				
Rarely	−0.796 (0.665)	−1.903 (0.617)*	−1.005 (0.643)	−3.207 (0.795)*
Sometimes	−0.645 (0.666)	−1.123 (0.613)	−0.358 (0.65)	− 1.793 (0.748)*
Often	−0.698 (0.697)	−1.206 (0.653)	− 0.97 (0.724)	− 1.766 (0.888)*
Always	−0.997 (0.708)	−1.736 (0.671)*	− 1.216 (0.78)	− 1.557 (1.177)
LTCQ7. Safe at home (Never)				
Rarely	−0.203 (1.396)	−1.136 (1.252)	− 0.105 (1.284)	− 0.596 (1.457)
Sometimes	−0.675 (1.323)	−1.88 (1.18)	− 1.025 (1.224)	− 0.978 (1.371)
Often	− 1.004 (1.319)	− 1.888 (1.173)	− 0.903 (1.218)	−1.094 (1.401)
Always	− 0.573 (1.323)	−1.758 (1.18)	−1.401 (1.233)	− 1.433 (1.41)
LTCQ8. Safe outside home (Never)				
Rarely	− 0.331 (0.551)	−0.432 (0.536)	− 0.35 (0.537)	0.309 (0.752)
Sometimes	−0.374 (0.526)	−0.258 (0.513)	− 0.47 (0.525)	0.135 (0.752)
Often	−0.373 (0.567)	− 0.442 (0.56)	− 0.675 (0.605)	0.252 (0.933)
Always	−0.751 (0.563)	− 0.595 (0.561)	−1.172 (0.637)*	−1.409 (1.145)
LTCQ10. Dependant (Always)				
Often	0.29 (0.345)	−0.050 (0.329)	−0.368 (0.348)	−1.664 (0.578)*
Sometimes	− 0.16 (0.338)	−0.715 (0.330)*	−1.278 (0.391)*	−2.034 (0.74)*
Rarely	0.22 (0.374)	−0.890 (0.394)*	− 0.939 (0.483)	− 15.902 (863.068)
Never	0.208 (0.377)	−1.286 (0.411)*	− 1.41 (0.567)*	−0.96 (1.069)
LTCQ11. Lonely (Always)				
Often	−0.301 (0.417)	− 0.083 (0.433)	0.447 (0.433)	0.723 (0.658)
Sometimes	−0.232 (0.415)	0.835 (0.430)	0.55 (0.466)	1.048 (0.699)
Rarely	0.075 (0.453)	1.126 (0.482)*	0.575 (0.562)	−12.693 (565.764)
Never	− 0.775 (0.418)	0.672 (0.457)	0.478 (0.535)	1.103 (0.958)
LTCQ12. Stigma (Always)				
Often	0.109 (0.514)	0.618 (0.499)	0.047 (0.504)	−0.667 (0.665)
Sometimes	0.819 (0.493)	1.057 (0.494)*	0.952 (0.5)	−0.035 (0.636)
Rarely	0.445 (0.504)	0.722 (0.511)	0.519 (0.544)	−1.073 (0.855)
Never	0.864 (0.51)	1.128 (0.520)*	1.356 (0.56)	−0.701 (0.909)
LTCQ15. Unhappy (Always)				
Often	0.463 (0.45)	0.140 (0.412)	−0.329 (0.409)	−1.389 (0.587)*
Sometimes	0.965 (0.463)*	−0.011 (0.437)	−1.007 (0.458)*	−1.479 (0.692)*
Rarely	0.71 (0.498)	−0.793 (0.494)	−2.147 (0.637)*	−2.276 (1.27)
Never	−0.045 (0.504)	−1.414 (0.502)*	− 1.878 (0.590)*	− 2.574 (1.326)
LTCQ19. Confident (Never)				
Rarely	−0.749 (0.711)	−0.479 (0.679)	− 0.15 (0.712)	−0.01 (0.868)
Sometimes	−0.97 (0.642)	−0.676 (0.622)	− 0.001 (0.659)	0.073 (0.83)
Often	0.136 (0.665)	−0.02 (0.654)	0.624 (0.708)	1.119 (0.967)
Always	−0.508 (0.654)	−0.602 (0.643)	− 0.067 (0.721)	− 1.796 (1.399)
Constant	1.786 (1.423)	3.638 (1.284)*	2.502 (1.341)	3.501 (1.451)*
**Anxiety and depression**				
LTCQ4. Control of daily life (Never)				
Rarely	1.227 (0.625)*	0.848 (0.597)	1.958 (0.683)*	1.642 (0.736)*
Sometimes	0.496 (0.563)	0.165 (0.53)	0.907 (0.64)	0.167 (0.759)
Often	0.563 (0.585)	0.016 (0.574)	0.708 (0.744)	0.162 (1.08)
Always	0.135 (0.598)	−1.225 (0.638)	− 1.833 (1.281)	0.743 (1.227)
LTCQ7. Safe at home (Never)				
Rarely	−2.398 (1.624)	−1.545 (1.606)	−0.767 (1.689)	−1.716 (1.694)
Sometimes	−2.161 (1.505)	−1.507 (1.523)	−0.94 (1.61)	−1.289 (1.603)
Often	−2.377 (1.474)	−1.526 (1.501)	−1.224 (1.596)	−2.486 (1.626)
Always	−2.495 (1.469)	−2.34 (1.5)	−1.682 (1.6)	− 2.846 (1.647)
LTCQ8. Safe outside home (Never)				
Rarely	0.996 (0.61)	1.558 (0.595)*	1.391 (0.663)*	1.701 (0.726)*
Sometimes	1.017 (0.522)	0.857 (0.526)	0.558 (0.605)	−0.316 (0.744)
Often	0.658 (0.538)	0.737 (0.57)	0.035 (0.746)	−1.057 (1.265)
Always	0.931 (0.543)	1.637 (0.593)*	0.987 (0.806)	0.249 (1.188)
LTCQ10. Dependant (Always)				
Often	−0.039 (0.341)	0.01 (0.363)	0.339 (0.445)	0.386 (0.547)
Sometimes	−0.098 (0.344)	0.116 (0.379)	0.49 (0.499)	0.103 (0.733)
Rarely	0.275 (0.386)	0.039 (0.47)	0.233 (0.704)	−0.571 (1.213)
Never	0.041 (0.402)	0.084 (0.531)	1.368 (0.812)	−0.174 (1.27)
LTCQ11. Lonely (Always)				
Often	0.912 (0.585)	−0.537 (0.544)	− 0.69 (0.598)	− 0.887 (0.689)
Sometimes	0.694 (0.531)	−1.033 (0.498)*	− 1.236 (0.582)*	− 1.826 (0.775)*
Rarely	0.501 (0.547)	−1.182 (0.537)*	−2.832 (0.904)*	−1.817 (1.018)
Never	−0.292 (0.536)	−1.721 (0.53)*	− 2.612 (0.79)*	− 2.31 (1.049)*
LTCQ12. Stigma (Always)				
Often	1.636 (0.675)*	1.297 (0.626)*	1.599 (0.683)*	1.797 (0.777)*
Sometimes	1.039 (0.575)	0.991 (0.523)	0.357 (0.612)	0.997 (0.738)
Rarely	1.12 (0.582)	0.289 (0.554)	−0.038 (0.7)	0.61 (0.891)
Never	0.891 (0.585)	0.598 (0.558)	0.794 (0.698)	1.756 (0.92)
LTCQ15. Unhappy (Always)				
Often	−0.439 (0.566)	−0.196 (0.54)	− 0.981 (0.582)	−1.431 (0.662)*
Sometimes	−0.807 (0.528)	−1.396 (0.522)*	−2.372 (0.607)*	−3.357 (0.858)*
Rarely	−1.525 (0.559)	−3.057 (0.64)*	−4.473 (1.178)*	− 3.361 (1.049)*
Never	−2.465 (0.586)	−2.705 (0.642)*	−4.287 (1.225)*	−16.266 (421.364)
LTCQ19. Confident (Never)				
Rarely	0.793 (0.843)	1.558 (0.819)	0.476 (0.894)	0.554 (0.934)
Sometimes	−0.016 (0.679)	0.778 (0.66)	0.196 (0.731)	0.207 (0.787)
Often	0.072 (0.691)	0.548 (0.686)	−0.163 (0.796)	−0.574 (1.005)
Always	−0.048 (0.69)	−0.141 (0.701)	− 0.978 (0.888)	−2.179 (1.41)
Constant	0.817 (1.527)	2.121 (1.517)	1.725 (1.604)	2.469 (1.588)

* *p*-value< 0.005

## Abbreviations

EQ-5D: EuroQoL 5 Dimension
LTCQ: Long-Term Conditions Questionnaire
OLS: Ordinary Least Square
PROM: Patient-reported outcome measure
LTC: Long-term conditions
LTCQ-8: Short-Form Long-Term Conditions Questionnaire
MAPS: MApping onto Preference-based measures reporting Standards
UK: United Kingdom
NICE: National Institute for Health and Care Excellence
COPD: Chronic Obstructive pulmonary disease
GP: General practitioner
DBIS: Disease Burden Impact Scale
CLAD: Censored least absolute deviations
HRQoL: Health-Related Quality of Life
SCRQoL: Social Care Related Quality of Life
ASCOT: Adult Social Care Outcomes Toolkit
GLM: Generalised Linear Model
MSE: Mean squared error
MAE: Mean absolute error
